# Increased C-Reactive Protein in Brazilian Children: Association with Cardiometabolic Risk and Metabolic Syndrome Components (PASE Study)

**DOI:** 10.1155/2019/3904568

**Published:** 2019-04-16

**Authors:** Lara Gomes Suhett, Helen Hermana Miranda Hermsdorff, Naruna Pereira Rocha, Mariane Alves Silva, Mariana De Santis Filgueiras, Luana Cupertino Milagres, Maria do Carmo Gouveia Peluzio, Juliana Farias de Novaes

**Affiliations:** Department of Nutrition and Health, Universidade Federal de Viçosa (UFV), Av. P.H. Rolfs s/n, Campus Universitário, CEP 36570-900 Viçosa, Minas Gerais, Brazil

## Abstract

C-reactive protein (CRP) is a marker of subclinical inflammation that has been found to be associated with cardiovascular disease risk. However, few studies have investigated the relationship between CRP and cardiometabolic markers in a representative sample of prepubescent children. The objective was to evaluate the high-sensitive CRP (hs-CRP) and its association with traditional and nontraditional cardiometabolic risk factors, as well as metabolic syndrome (MetS) components in Brazilian children. This is a cross-sectional representative study, with participants of the Schoolchildren Health Assessment Survey (PASE). Children from 8 to 9 years old (*n*=350) enrolled in public and private schools in the municipality of Viçosa, Minas Gerais, Brazil, were evaluated. Sociodemographic evaluation was performed through a semistructured questionnaire. Anthropometric, body composition, clinical, and biochemical measures were analyzed for cardiometabolic risk assessment. The total mean of serum hs-CRP concentration was 0.62 (±1.44) mg/L. hs-CRP was significantly correlated with several anthropometric, biochemical, and clinical parameters in this population (*P* < 0.05). hs-CRP was positively associated with the accumulation of cardiometabolic risk factors and MetS components (*P* < 0.05). Children with excessive weight; abdominal obesity; increased gynoid and android body fat; low HDL-c; hyperglycemia; and elevated uric acid, homocysteine, and apoB had higher chances of presenting increased hs-CRP (*P* < 0.05). In this study, Brazilian children with cardiometabolic risk already presented elevated serum hs-CRP concentration. hs-CRP was associated with the increase of traditional and nontraditional cardiometabolic risk factors, as well as the accumulation of MetS components.

## 1. Introduction

In the last decades, overweight and obesity prevalence has increased considerably in pediatric populations worldwide and has become a serious public health problem [1, 2]. This is worrisome since overweight, the accumulation of body fat [3], might be associated with subclinical inflammation [4, 5] because the adipose tissue can act as an endocrine organ and produce several proinflammatory cytokines, such as interleukin-6 (IL-6), tumor necrosis factor (TNF-alpha), and C-reactive protein (CRP) [6].

The CRP is an acute phase plasma protein used to diagnose chronic inflammatory diseases, and it is related to the development of cardiovascular disease (CVD) and atherosclerosis [7, 8]. Also, evidences suggest that moderated elevations in serum CRP concentration can independently predict cardiometabolic changes in adults, as well as endothelial lesions [9–12].

Unlike studies with adults, the results found with children are less likely to be affected by chronic conditions. Therefore, studies with the infant population could contribute to the best understanding of the relationship between obesity, hyperglycemia, dyslipidemia, metabolic syndrome (MetS), and subclinical inflammation.

In the last years, the number of clinical studies exploring the subclinical inflammation and its associated factors during childhood has considerably increased aiming to identify children at risk of later CVD. Some studies have shown a positive correlation between CRP and anthropometric parameters, such as body mass index (BMI) [4, 5, 13] and waist circumference [14–16], in children. However, results are still controversial [17, 18], and few studies have investigated the relationship between this protein with nontraditional cardiometabolic markers and MetS components in a representative sample.

Due to the scarcity of research evaluating the relationship between subclinical inflammation and cardiometabolic risk with prepubertal children, the objective of this study was to evaluate the serum high-sensitive CRP (hs-CRP) and its association with traditional and nontraditional cardiometabolic risk factors, as well as MetS components in Brazilian children.

## 2. Materials and Methods

### 2.1. Participants and Study Design

This study is part of the Schoolchildren Health Assessment Survey (PASE, *Pesquisa de Avaliação da Saúde do Escolar*), which is a cross-sectional representative research with the objective to evaluate cardiovascular health in children from the city of Viçosa, Minas Gerais (MG), Brazil.

The municipality of Viçosa, located in the Zona da Mata Mineira region, has a territorial extension of 299.4 km^2^ and 72.220 inhabitants, with 93.2% of the population residing in urban areas, according to the 2015 Census [19].

In this study, the sample consisted of 350 children, ages 8 and 9, enrolled in all public and private schools in the urban area of Viçosa.

Children with hs-CRP ≥ 10 mg/L (*n*=28) were excluded from the study once hs-CRP concentrations above this value are associated with inflammation and more severe infections, and not to subclinical inflammation. More information about the study design, sample calculation, and participants has already been described in previously published PASE study [20].

### 2.2. Sociodemographic and Lifestyle Data

A semistructured questionnaire was applied to assess the following sociodemographic, economic, and lifestyle variables: sex, age, ethnicity, family income per month (R$, *reais*), and screen time per day (hours spent watching television, playing video game, and using cellphone or computer) of the child. Children were classified with sedentary behavior when screen time was more than 2 h/day [21]. All data were answered by parents or guardians. All these variables were used for adjustments in the final regression model.

### 2.3. Anthropometric, Body Composition, and Clinical Data

Trained nutritionists performed anthropometric evaluations. Weight and height were measured according to the standards recommended by Jelliffe [22], using, respectively, a digital electronic scale with a capacity of 150 kg and a sensitivity of 100 g (Tanita® Ironman Model BC 553, Tanita Corporation of America Inc, Artlington Heights, USA) and a vertical stadiometer divided in centimeters and subdivided in millimeters (Alturexata®, Belo Horizonte, Brazil). We calculated the body mass index (BMI) with the data obtained from these measurements.

The nutritional status of the child was determined through BMI-for-age cutoff points by *z*-score on a WHO AnthroPlus software [23] and classified according to the World Health Organization [24]. Children who were overweight or obese were classified as having excessive weight.

Waist and neck circumference was measured using an inelastic tape (TBW®, SP, Brazil) divided in centimeters and subdivided in millimeters. Waist circumference (WC) measurements started at the midpoint between the iliac crest and the last rib, and the neck circumference (NC) was assessed at the level of the thyroid cartilage. Abdominal obesity and increased NP were classified according to the cutoff points proposed by Filgueiras et al. [25] and Nafiu et al. [26], respectively.

The hypertriglyceridemic waist phenotype (HTGWP) was classified by the simultaneous presence of increased WC and hypertriglyceridemia [27].

The waist-to-height ratio (WHtR) was calculated by dividing WC by height. The cutoff point WHtR ≥0.5 was used as a risk factor for the development of cardiometabolic diseases [28].

Body composition evaluation was performed using the dual-energy X-ray absorptiometry (DXA) method (Lunar Prodigy Advance, GE Medical Systems Lunar, Milwaukee, WI, USA). The examination was performed in the diagnostic imaging sector of the UFV Health Center by a specialized technician. During the examination, the child had fasted and was wearing light clothing without any metal trim. The child remained supine on a stretcher until the reading was completed by the equipment. Excessive body fat was assessed using the cutoff points proposed by Lohman [29], and the increased gynoid and android body fat were classified using the 85th percentile of the sample.

A previously trained team measured blood pressure using an automatic device (Omron® HEM 907 Veron Hills, Illinois, USA). Each child sat at rest for 5 minutes with their right arm at the same level as their heart. Subsequently, blood pressure was measured three times, and the mean of the last two measurements was evaluated according to the Brazilian Hypertension Society [30].

### 2.4. Biochemical Variables

Biochemical examinations were performed at the Clinical Analysis Laboratory of the UFV Health Center. Blood samples were collected by venipuncture at the antecubital region of children who had gone through 12 hours of fasting. Aliquots of this biological material were kept in 2 ml Eppendorf, encoded, and stored at −80°C. We measured total cholesterol concentration and fractions (high-density lipoprotein (HDL-c) and low-density lipoprotein (LDL-c)), as well as concentrations of triglycerides, glucose, uric acid, and high-sensitive C-reactive protein (hs-CRP) using automated equipment (BioSystems 200 Mindray® model, Nanchan, China), according to the manufacturer recommendations in the Bioclin® kits (Belo Horizonte, MG, Brazil). hs-CRP and uric acid concentrations were measured by an immunoturbidimetric and enzymatic colorimetric methods, respectively.

Total cholesterol ≥170 mg/dL, HDL-c <45 mg/dL, LDL-c ≥110 mg/dL, triglycerides ≥75 mg/dL, glucose ≥100 mg/dL, and fasting insulin >15 *μ*U/ml were considered inadequate according to the Brazilian Cardiology Society (2017) [31] and the American Diabetes Association (2017) [32]. Due to the absence of cutoff points defined for the age range of the study, the 90th and the 85th percentiles of the sample were used to classify increased values of hs-CRP (≥1.82 mg/L) and uric acid, respectively.

The insulin electrochemiluminescence immunoassay was done to evaluate insulin using the Elecsys Insulin® test by Elecsys Insulin® test (Roche Diagnostics, Indianapolis, IN, USA) with a detection limit of 0.200–1000 *μ*U/mL. Insulin resistance was estimated by the homeostasis model of insulin resistance (HOMA-IR) according to Matthews et al. [33], and increased values were classified according to the 85th percentile of the sample.

The serum homocysteine, leptin, and apolipoproteins A1 (ApoA1) and B (apoB) concentrations were analyzed through commercials ELISA kits, using the chemiluminescence method (standardized protocols from Diagnóstico Brasil), enzyme immunoassay method, and kinetic nephelometry method (Beckman Coulter, CA, USA), respectively. Due to the lack of specific cutoff points for children, altered values were classified using the 15th percentile for ApoA1 and the 85th percentiles for homocysteine, leptin, and apoB. The increased apoB/ApoA1 ratio was also calculated using as cutoff point the 85th percentile of the sample [34].

### 2.5. Statistical Analysis

Analysis was carried out in the Statistical Package for the Social Sciences® (SPSS) version 21 and Stata version 13 (StataCorp LP). The Kolmogorov–Smirnov test was used to evaluate the normality of the variables, as well as graphical analysis and asymmetry coefficients. The Pearson chi-square test was used to verify association between categorical variables. Spearman's correlation coefficient was used to evaluate the association between serum hs-CRP and the independent variables.

Increased hs-CRP showed a prevalence of 10% in the sample; therefore, the results were presented as odds ratio (OR) and 95% confidence interval (CI 95%) measured by logistic regression [35, 36]. Analyses were performed between the concentrations of hs-CRP (dependent variable) and anthropometric, body composition, biochemical, and clinical variables (explanatory variables). Sex, age, ethnicity, income, sedentary behavior, and body fat percentage were used as adjustment variables in the final regression models. The Hosmer–Lemeshow test showed the fit of the final model to be adequate (*P* > 0.05).

In addition, the association between hs-CRP and numbers of cardiometabolic risk factors was verified through a simple linear regression adjusting for age, sex, ethnicity, income, sedentary behavior, and body fat percentage. Traditional cardiometabolic risk factors (excess of weight; increased WP, NP, % body fat, gynoid and android body fat, serum total cholesterol, LDL-c, triglycerides, HOMA-IR, insulin, and blood pressure; low HDL-c; WHtR >0.5; and hyperglycemia); metabolic syndrome (MetS) components (increased WP, blood pressure, glucose, triglycerides, and low HDL-c); nontraditional cardiometabolic risk factors (increased uric acid, homocysteine, leptin, apoB; low ApoA1; and presence of HTGWP); and accumulation of cardiometabolic risk factors from the final regression model. The level of statistical significance was 5%.

### 2.6. Ethical Standard Disclosure

This study was conducted according to the guidelines laid down in the Declaration of Helsinki, and all the procedures involving human subjects were approved by the Ethics Committee on Human Research of the Federal University of Viçosa (UFV), case no. 663.171/2014. Also, this project was presented to the Municipal Department of Education, the Regional Superintendent of Education, and principals of schools. Written informed consent was obtained from all the children's parents after the researchers read and explained the document.

## 3. Results

Approximately half of the sample was 9 years old (51.4%) and female (52.6%). A considerable part of the sample was nonwhite (67.4%). High prevalence of excessive weight (32%) and body fat (48.3%) were observed (Table 1). The total mean of serum hs-CRP concentration was 0.62 (±1.44) mg/L.

There was a higher prevalence of increased hs-CRP (≥90th percentile) in overweight children (15.2%), with increased WP (19.7%), elevated gynoid (20.8%), android (21.2%) body fat, low HDL-c (17.2%), and hyperglycemia (50.0%) (Table 1). There was also a higher prevalence of increased hs-CRP in children with the presence of nontraditional cardiometabolic risk factors such as increased concentrations of uric acid (23.7%), homocysteine (20.5%), leptin (18.9%), and apoB (23.1%) (Table 2).

In addition, we found that hs-CRP was correlated with several anthropometric, biochemical, and clinical parameters in this population (*P* < 0.05) (Table 3).

According to the multivariate logistic regression analysis, children with excessive weight, abdominal obesity (OR: 2.90, 95% CI: 1.37–6.13), increased gynoid (OR: 2.80, 95% CI: 1.23–6.43) and android body fat (OR: 2.91, 95% CI: 1.26–6.72), low HDL-c (OR: 2.60, 95% CI: 1.23–5.50), hyperglycemia (OR: 14.23, 95% CI: 2.57–78.68), increased uric acid (OR: 3.59, 95% CI: 1.60–8.02), homocysteine (OR: 2.81, 95% CI: 1.08–7.36), and apoB (OR: 2.84, 95% CI: 1.09–7.40) presented higher chances to have increased hs-CRP regardless of age, sex, ethnicity, income, sedentary behavior, and body fat percentage (Table 4).

**Figure 1:**
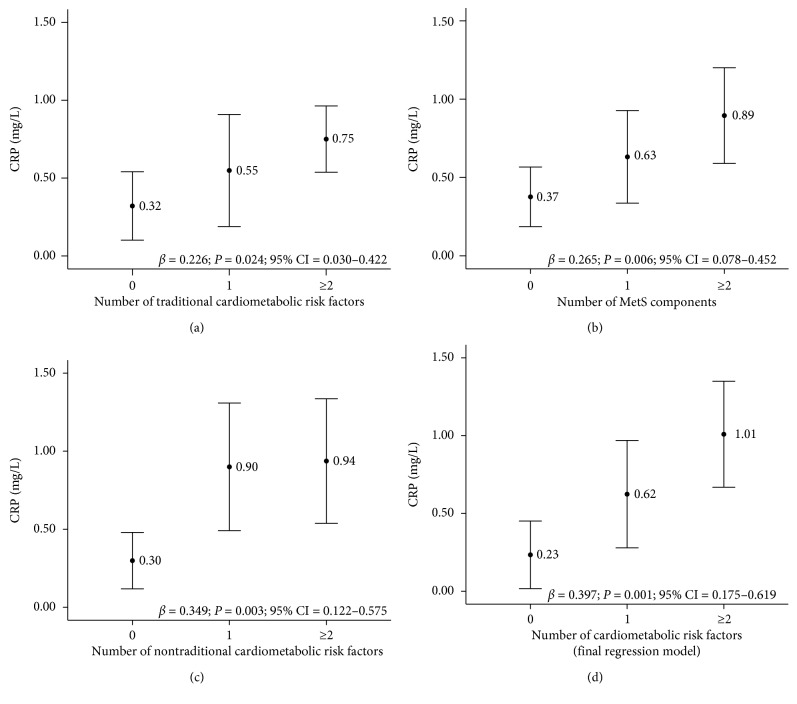
Mean serum hs-CRP concentration (dependent variable) according to the accumulation of altered (a) traditional cardiometabolic risk factors, (b) metabolic syndrome components, (c) nontraditional cardiometabolic risk factors, and (d) risk factors from the final regression model in children, Viçosa, MG, Brazil, 2015. Multiple linear regression (^*∗*^*P* < 0.05) adjusted for age, sex, ethnicity, income, and sedentary behavior.

We emphasize the associations between hs-CRP and the accumulation of traditional cardiometabolic risk factors (*β* = 0.226; *P*=0.024; 95% CI = 0.030–0.422), MetS components (*β* = 0.265; *P*=0.006; CI 95% = 0.078–0.452), nontraditional cardiometabolic risk factors (*β* = 0.349; *P*=0.003; CI 95% = 0.122–0.575), and cardiometabolic risk factors identified in the final regression model presented in Table 4 (*β* = 0.397; *P*=0.001; CI 95% = 0.175–0.619) (Figure 1).

## 4. Discussion

In the present study, children with traditional cardiometabolic risk factors (excessive weight and increased gynoid and android body fat), with MetS components (abdominal obesity, low HDL-c, and hyperglycemia), and nontraditional cardiometabolic risk factors (increased uric acid, homocysteine, and apoB) presented higher chances to have elevated hs-CRP. Serum hs-CRP concentrations were positively associated with the increase in the number of cardiometabolic risk factors and MetS components.

The association of serum hs-CRP with excessive weight and abdominal obesity is consistent with previous studies performed with children and adolescents classified as overweight and obese by the BMI or body fat percentage [4, 5, 13, 16, 37], as well as in children with increased waist circumference [13–15, 38]. This association suggests that, although there are no cutoff points of hs-CRP for infant population, obesity-related subclinical inflammation is already observed in early ages. This result can be explained by the increase in interleukin-6 (IL-6) levels, the main regulator of CRP synthesis in the liver, in individuals with excessive weight and increased central adiposity once this cytokine is secreted by adipocytes [8, 15, 39]. Moreover, research has shown that childhood obesity and the presence of subclinical inflammation may promote the activation of atherosclerotic mechanisms [15, 40]. We believe that increased hs-CRP might be a potent mediator between the obesity and the beginning of atherosclerosis in childhood.

The relationship between lipid metabolism and systemic inflammation has already been evidenced [41, 42]. In this study, we also observed an association between hs-CRP and low HDL-c. Muramoto et al. [42], in a research conducted with 124 children and adolescents, observed that individuals with CRP ≥5 mg/L presented changes in the lipid profile, and, for each increase of 1 mg/L in serum CRP concentration, a reduction of 0.072 mg/dL HDL-c occurs, regardless of the individual's nutritional status. Other researches with children and adolescents also observed equivalent results [5, 14, 43, 44]. Some predisposed individuals with high body fat can produce different cytokines in the adipose tissue that could mediate the association between hs-CRP and HDL-c [16]. Another possible mechanism could be related to alterations in IL-6 levels, as this adipokine stimulates lipolysis by affecting the action of lipoprotein lipase [45].

Furthermore, we found an association between serum hs-CRP and hyperglycemia. It is known that subclinical inflammation and activated innate immunity have an important role in the pathophysiology of abnormal glucose metabolism [46, 47]. Studies with adults have shown risk of type 2 diabetes mellitus (DM2) development to be highly associated with increased CRP, suggesting that low-grade chronic inflammation coexists with glucose intolerance and compensatory increase in insulin secretion [48, 49]. Therefore, it is indicated that this inflammatory marker can be a powerful predictor of DM2 [47] and may be used to better target individuals for lifestyle interventions [46–50].

Although the mechanisms underlying the association between CRP and hyperglycemia have not been fully elucidated, there are some possible explanations. This association may be in part linked to an increase production of proinflammatory cytokines, such as IL-6 and TNF-*α* [46, 47]. These proinflammatory markers, as well as CRP, are suggested to induce insulin resistance and gluconeogenesis, subsequent hyperglycemia, and attenuate insulin signaling and sensitivity through insulin receptor substrate phosphorylation [46–50]. Moreover, hyperglycemia was the best predictor to hs-CRP in this population; however, further colongitudinal investigations with the pediatric population are necessary to better elucidate the mechanisms involved in this relationship.

In this study, hs-CRP was associated with nontraditional cardiometabolic risk factors. There are evidences showing that hyperuricemia, hyperhomocysteinemia, and increased atherogenic apoB are involved in the pathogenesis of endothelial dysfunction and atherosclerosis process by inducing proliferation of vascular smooth muscle cells, increasing thromboxane formation, impairing nitric oxide production, stimulating oxidative stress, and inducing vascular inflammation and artery damage [51–53]. Researches have demonstrated that uric acid stimulates the production of IL-6, TNF-*α*, and up-regulates CRP expression by human mononuclear cells and cultured human vascular cells, respectively [51, 54].

Our results are similar to previous studies linking hs-CRP with uric acid [54–56], homocysteine [57, 58], and apoB concentrations [55] in children, suggesting that these altered markers may be associated with the development of subclinical inflammation and aggravate the risk of CVD. However, studies are controversial [38, 59], and there is still a gap in the knowledge on the relationship between hs-CRP and nontraditional risk factors in infant population.

Studies with adults have shown an association between serum hs-CRP concentration and increased numbers of cardiometabolic risk factors [11, 60]. In addition, Giannini et al. [16] evaluated the association between hs-CRP and MetS in Brazilian adolescents, identifying that this marker was progressively higher in those with higher numbers of MetS components. Similar results were found in other studies conducted with Brazilian [61] and Chilean youth [62], suggesting the possibility of using hs-CRP as maker of MetS [16, 62]. Our results show that this association is also observed in the beginning of life, corroborating with the data presented by Guran et al. [63]. Moreover, this is the first study to evaluate the association between hs-CRP and the accumulation of nontraditional cardiometabolic risk factors in a representative pediatric population.

Although the consequences of subclinical inflammation in children are not fully understood, studies have shown increased serum hs-CRP concentration to lead to adverse effects on vascular endothelium, which, together with cardiometabolic alterations, may favor the development of atherosclerosis [63–65]. In this study, we observed a prevalence of 9.1% (*n*=32) of the evaluated children to have hs-CRP levels greater than 2 mg/L, considered to be a CVD high-risk factor in adult population [10, 31]. Thus, children with increased hs-CRP levels, especially those with excessive weight, need early prevention strategies to avoid the emergence of associated comorbidities.

Our study had some limitations. First is the absence of a well-established cutoff point to define high values for hs-CRP in children. However, our results are similar to other studies with adults and adolescents [10, 16, 31], and therefore, the cutoff of 2 mg/L to establish associations with cardiometabolic risk factors and MetS components in the infant population seem to be also reasonable, as shown in our study (≥90 percentile = 1.82 mg/L). Second is the use of a single measurement of hs-CRP. It is recommended to repeat the measurements once concentrations might be affected by recent inflammation and more severe infections; nevertheless, children with CRP ≥10 mg/L were excluded from the study. Third, measurement of other markers of inflammation could provide further information on the association between subclinical inflammation and cardiometabolic risk factors in infancy. Lastly, we emphasize the cross-sectional nature of our study, which does not allow for establishing causal relationship between hs-CRP and risk factors.

Some benefits of this study should be considered. The sample was homogeneous in relation to the physiological characteristics, being constituted by prepubertal children, which contributes to the reduction of possible influences on body composition. Moreover, this research is one of the few conducted in developing countries that investigated the association between serum hs-CRP concentration and cardiometabolic risk factors in childhood, being the first Brazilian representative study to date with prepubescent children. As metabolic changes influence subclinical inflammation and vice versa, the evaluation of hs-CRP can be considered a useful tool for the early detection of children at higher risk of developing CVD and MetS.

## 5. Conclusion

In conclusion, Brazilian children presented higher chances to have increased hs-CRP in the presence of traditional cardiometabolic risk factors (excessive weight and elevated gynoid and android body fat), MetS components (abdominal obesity, low HDL-c, and hyperglycemia), and nontraditional cardiometabolic risk factors (increased uric acid, homocysteine, and apoB), indicating the early occurrence of metabolic disorders and their relation to subclinical inflammation. Serum hs-CRP concentration was also directly associated with an increase in the number of cardiometabolic risk factors (traditional and nontraditional) and MetS components. Since hs-CRP assessment is a low-cost test and an important marker during the acute phase of inflammatory processes, we suggest that this protein should be routinely evaluated in children with similar characteristics of this population aiming the prevention of CVD and MetS in the future.

## Figures and Tables

**Table 1:** Traditional cardiometabolic risk factors of children according to serum hs-CRP concentration, Viçosa, MG, Brazil, 2015.

Cardiometabolic risk factors	Total		hs-CRP (mg/L)				*P* values
			<P90		≥P90		
	*n*	%	*n*	%	*n*	%	
BMI *z*-score (kg/m^2^)							0.027^*∗*^
** **Normal	238	68.0	220	92.4	18	7.6	
** **Overweight/obese	112	32.0	95	84.8	17	15.2	

↑ Waist circumference (cm)							0.001^*∗*^
** **Absent	274	78.3	245	92.7	20	7.3	
** **Present	76	21.7	61	80.3	15	19.7	

↑ Neck circumference (cm)^†^							1.000
** **Absent	324	92.6	291	89.8	33	10.2	
** **Present	26	7.4	24	92.3	2	7.7	

↑ WHtR							0.125
** **Absent	292	83.43	266	91.1	26	8.9	
** **Present	58	16.57	49	84.5	9	15.5	

↑ Body fat (%)							0.144
** **Absent	181	51.7	167	92.3	14	7.7	
** **Present	169	48.3	148	87.6	21	12.4	

↑ Gynoid body fat (g)^‡^							0.005^*∗*^
** **Absent	297	84.9	273	91.9	24	8.1	
** **Present	53	15.1	42	79.2	11	20.8	

↑ Android body fat (g)^‡^							0.004^*∗*^
** **Absent	298	85.1	274	91.9	24	8.1	
** **Present	52	14.9	41	78.8	11	21.2	

↑ TC (mg/dL)							0.576
** **Absent	273	78.0	247	90.5	26	9.5	
** **Present	77	22.0	68	88.3	9	11.7	

↓ HDL-c (mg/dL)							0.005^*∗*^
** **Absent	251	71.7	233	92.8	18	7.2	
** **Present	99	28.3	82	82.8	17	17.2	

↑ LDL-c (mg/dL)							0.616
** **Absent	299	85.7	270	90.3	29	9.7	
** **Present	50	14.3	44	88.0	6	12.0	

↑ Triglyceride (mg/dL)							0.300
** **Absent	189	54.0	173	91.5	16	8.5	
** **Present	161	46.0	142	88.2	19	11.8	

↑ Glucose (mg/dL)^†^							0.015^*∗*^
** **Absent	343	98.3	311	90.7	32	9.3	
** **Present	6	1.7	3	50.0	3	50.0	

↑ HOMA-IR^‡^							0.166
** **Absent	296	85.0	269	90.9	27	9.1	
** **Present	52	15.0	44	84.6	8	15.4	

↑ Insulin (*μ*U/ml)^†^							0.473
** **Absent	342	98.3	308	90.1	34	9.9	
** **Present	6	1.7	5	83.3	1	16.7	

↑ Blood pressure (mmHg)^†^							0.237
** **Absent	314	90.0	295	93.9	19	6.1	
** **Present	35	10.0	35	100.0	0	0.0	

hs-CRP, high-sensitive C-reactive protein; WHtR, waist-to-height ratio; HOMA-IR, homeostasis model assessment of insulin resistance; HDL-c, high-density lipoprotein; LDL-c, low-density lipoprotein; TC, total cholesterol. ^‡^Classification according to the 85th percentile of the sample. Pearson's chi-square test (^*∗*^*P* < 0.05); ^**†**^Fisher's exact test (^*∗*^*P* < 0.05).

**Table 2:** Nontraditional cardiometabolic risk factors of children according to serum hs-CRP concentration, Viçosa, MG, Brazil, 2015.

Cardiometabolic risk factors	Total		hs-CRP (mg/L)				*P* values
			<P90		≥P90		
	*n*	%	*n*	%	*n*	%	
↑ Uric acid (mg/dL)^‡^							<0.001^*∗*^
** **Absent	291	83.1	270	92.8	21	7.2	
** **Present	59	16.9	45	76.3	14	23.7	

↑ Homocysteine (ng/mL)^‡^							0.033^*∗*^
** **Absent	221	85.0	201	91.0	20	9.0	
** **Present	39	15.0	31	79.5	8	20.5	

HTGWP							0.143
** **Absent	299	85.4	272	91.0	27	9.0	
** **Present	51	14.6	43	84.3	8	15.7	

↑ Leptin (ng/mL)^‡^							0.021^*∗*^
** **Absent	294	84.7	268	90.2	29	9.8	
** **Present	53	15.3	43	81.1	10	18.9	

↓ ApoA1 (mg/dL)^‡‡^							0.694
** **Absent	297	84.8	268	90.5	28	9.5	
** **Present	52	15.2	46	88.5	6	11.5	

↑ apoB (mg/dL)^‡^							0.007^*∗*^
** **Absent	221	85.0	202	91.4	19	8.6	
** **Present	39	15.0	30	76.9	9	23.1	

↑ apoB/ApoA1^‡^							0.318
** **Absent	220	84.9	198	90.0	22	10.0	
** **Present	39	15.1	33	84.6	6	15.4	

hs-CRP, high-sensitive C-reactive protein; HTGWP, hypertriglyceridemic waist phenotype; apoB, apolipoprotein B; ApoA1, apolipoprotein A1. Classification according to the 15th^‡‡^ and 85th^‡^ percentiles of the sample. Pearson's chi-square test (^*∗*^*P* < 0.05).

**Table 3:** Spearman's correlation between serum hs-CRP concentration and anthropometric, biochemical, and clinical parameters in children, Viçosa, MG, Brazil, 2015.

Variables	hs-CRP (mg/L)	
	*r*	*P* values
BMI (kg/m^2^)	0.191	<0.001^*∗*^
Waist circumference (cm)	0.217	<0.001^*∗*^
Neck circumference (cm)	0.173	0.001^*∗*^
WHtR	0.228	<0.001^*∗*^
Body fat (%)	0.206	<0.001^*∗*^
Gynoid body fat (g)	0.222	<0.001^*∗*^
Android body fat (g)	0.195	<0.001^*∗*^
TC (mg/dL)	−0.031	0.561
HDL-c (mg/dL)	−0.095	0.076
LDL-c (mg/dL)	−0.019	0.720
Triglyceride (mg/dL)	0.018	0.735
Glucose (mg/dL)	0.048	0.372
HOMA-IR	0.163	0.002^*∗*^
Insulin (*μ*U/ml)	0.170	0.001^*∗*^
Diastolic BP (mmHg)	0.100	0.061
Systolic BP (mmHg)	0.110	0.041^*∗*^
Uric acid (mg/dL)	0.101	0.060
Homocysteine (ng/mL)	0.099	0.110
HTGWP	0.150	0.005^*∗*^
Leptin (ng/mL)	0.033	0.534
ApoA1 (mg/dL)	−0.071	0.187
apoB (mg/dL)	0.045	0.471
apoB/ApoA1	0.076	0.222

hs-CRP, high-sensitive C-reactive protein; WHtR, waist-to-height ratio; HOMA-IR, homeostasis model assessment of insulin resistance; HDL-c, high-density lipoprotein; LDL-c, low-density lipoprotein; TC, total cholesterol; BP, blood pressure; HTGWP, hypertriglyceridemic waist phenotype; apoB, apolipoprotein B; ApoA1, apolipoprotein A1. Spearman's correlation test (^*∗*^*P* < 0.05).

**Table 4:** Crude and adjusted odds ratio (OR) of the association between serum hs-CRP concentrations and cardiometabolic risk factors in children, Viçosa, MG, Brazil, 2015.

Cardiometabolic risk factors	hs-CRP ≥ P90 (dependent variable)			
	Crude		Adjusted	
	OR (95% CI)	*P* value	OR (95% CI)	*P* value
Traditional				
** **Overweight/obese^1^	2.19 (1.08–4.43)	0.030^*∗*^	2.08 (1.01–4.29)	0.046^*∗*^
** **↑ Gynoid body fat^1^	2.98 (1.36–6.52)	0.006^*∗*^	2.80 (1.23–6.43)	0.014^*∗*^
** **↑ Android body fat^1^	3.06 (1.39–6.72)	0.005^*∗*^	2.91 (1.26–6.72)	0.012^*∗*^

MetS components				
** **↑ Waist circumference^1^	3.12 (1.51–6.45)	0.002^*∗*^	2.90 (1.37–6.13)	0.005^*∗*^
** **↓ HDL-c^2^	2.68 (1.32–5.45)	0.006^*∗*^	2.60 (1.23–5.50)	0.012^*∗*^
** **↑ Glucose^2^	9.74 (1.89–50.32)	0.007^*∗*^	14.23 (2.57–78.68)	0.002^*∗*^

Nontraditional				
** **↑ Uric acid^2^	4.00 (1.89–8.43)	<0.001^*∗*^	3.59 (1.60–8.02)	0.002^*∗*^
** **↑ Homocysteine^2^	2.59 (1.05–6.40)	0.039^*∗*^	2.81 (1.08–7.36)	0.034^*∗*^
** **↑ apoB^2^	3.19 (1.32–7.70)	0.010^*∗*^	2.84 (1.09–7.40)	0.033^*∗*^

hs-CRP, high-sensitive C-reactive protein; BMI, body mass index; HDL-c, high-density lipoprotein; apoB, apolipoprotein B; CI, confidence interval. Multivariate logistic regression (^*∗*^*P* < 0.05), using as reference hs-CRP < P90 (<1.82 mg/L). ^1^Adjusted by age, sex, ethnicity, income, and sedentary behavior; ^2^adjusted by model 1 + body fat percentage.

## Data Availability

The data used to support the findings of this study are available from the corresponding author upon request.
